# Cathelicidin Peptides Restrict Bacterial Growth via Membrane Perturbation and Induction of Reactive Oxygen Species

**DOI:** 10.1128/mBio.02021-19

**Published:** 2019-09-10

**Authors:** Dean A. Rowe-Magnus, Adenine Y. Kao, Antonio Cembellin Prieto, Meng Pu, Cheng Kao

**Affiliations:** a Biology Department, Indiana University, Bloomington, Indiana, USA; b Department of Molecular and Cellular Biochemistry, Indiana University, Bloomington, Indiana, USA; University of British Columbia

**Keywords:** cathelicidins, ROS, antimicrobial peptides, biofilms, Gram-negative bacteria, single-cell

## Abstract

All metazoans produce antimicrobial peptides (AMPs) that have both broad antimicrobial and immunomodulatory activity. Cathelicidins are AMPs that preferentially kill Gram-negative bacteria *in vitro*, purportedly by assembling into higher-order structures that perforate the membrane. We utilized high-resolution, single-cell fluorescence microscopy to examine their mechanism of action in real time. Engineered cathelicidins rapidly bound to Gram-negative and Gram-positive cells and penetrated the cytoplasmic membrane. Rapid failure of the peptidoglycan superstructure in regions of active turnover caused leakage of cytoplasmic contents and the formation of membrane-bound blebs. A mutation anticipated to destabilize interactions between cathelicidin subunits had no effect on bactericidal activity, suggesting that cathelicidins have activities beyond perforating the membrane. Nanomolar concentrations of cathelicidins, although not bactericidal, reduced the growth rate of Gram-negative and Gram-positive bacteria. The cells exhibited expression changes in multiple essential processes, including protein synthesis, peptidoglycan biosynthesis, respiration, and the detoxification of reactive oxygen species (ROS). Time-lapse imaging revealed that ROS accumulation preceded bleb formation, and treatments that reduced cellular ROS levels overcame these bactericidal effects. We propose that that the primary effect of cathelicidins is to induce the production of ROS that damage bacterial molecules, leading to slowed growth or cell death. Given their low circulating levels *in vivo*, AMPs may serve to slow bacterial population expansion so that cellular immunity systems can respond to and battle the infection.

## Abstract (precis)

Antimicrobial peptides (AMPs) are an important part of the mammalian innate immune system in the battle against microbial infection. How AMPs function to control bacteria is not clear, as nearly all activity studies use nonphysiological levels of AMPs. We monitored peptide action in live bacterial cells over short time frames with single-cell resolution and found that the primary effect of cathelicidin peptides is to increase the production of oxidative molecules that cause cellular damage in Gram-positive and Gram-negative bacteria.

## INTRODUCTION

All metazoans produce antimicrobial peptides (AMPs) that serve as a part of the first line of defense against infection by bacteria, viruses, and fungi (1, 2). The cathelicidin family is a large and diverse collection of peptides that are released from mammalian cells by proteolysis of cathelin molecules in response to microbial infections (3). The solubilized peptides, typically 25 to 45 amino acids in length, contain an amphipathic α-helix with a high abundance of basic amino acids, followed by a less structured sequence. Cathelicidins are thought to interact with each other within the membranes of susceptible bacteria to form assemblages that affect permeability and result in cell death (4). Notably, relatively few resistance mechanisms have evolved against AMPs.

The human cathelicidin LL-37 has important roles in modulating inflammatory responses. LL-37 can sequester lipopolysaccharides (LPS) to decrease signaling by Toll-like receptor 4 (TLR4) (5, 6), and it has been demonstrated to prevent sepsis in animal models (7). Perhaps because they are released in response to bacterial infection and tissue injuries, LL-37 and other cathelicidins can promote wound healing and reduce fibrosis (8–10). All of these desirable activities have generated significant interest in developing cathelicidins to treat bacterial infection and injuries, however, concerns exist for their use. First, sustained and elevated levels of LL-37 can trigger hyperinflammation by binding nucleic acids and inducing receptor-mediated endocytosis that activates Toll-like receptor 3 to trigger proinflammatory responses (11, 12). Elevated LL-37 levels are also associated with autoimmune diseases such as lupus and psoriasis (8, 9), and bacterial killing requires concentrations that are orders of magnitude higher than those present in human plasma (13). In order to improve the activity of cathelicidins and to differentially alter one or more of its numerous activities, we previously demonstrated that truncated LL-37 derivatives with an increased overall positive charge exhibited reduced activation of TLR3 signaling while increasing bactericidal activity (10). Shorter, positively charged cathelicidins produced by other mammals also exhibit reduced activation of proinflammatory responses, increased bactericidal activity, and reduced lysis of human red blood cells compared to LL-37. A peptide from cows, BMAP-27B, was found to rapidly kill Gram-negative bacteria that were resistant to polymyxins (10). Like LL-37, BMAP-27B was poor at killing Gram-positive bacteria, requiring ca. 10-fold-higher concentrations to reach the MICs.

The engineering of therapeutically useful cathelicidins will require an understanding of their mechanism of action. Most mechanistic studies of the bacteriostatic and bactericidal effects of cathelicidins and other AMPs use bulk, planktonic cultures. Although bulk assays provide valuable insight into the consequences of AMP activity, initial damage can occur more quickly than the response time of typical bulk measurements. Moreover, cell heterogeneity and subcellular spatial information cannot be gleaned from bulk assays. How cathelicidins affect cells at physiologically relevant levels is also unclear, since they are usually tested *in vitro* at concentrations that far exceed typical physiological levels (∼50 ng/ml for LL-37) (13, 14). A clearer understanding of AMP killing mechanics and kinetics should aid in the design of AMPs for therapeutic use (15). Here, we use single-cell, real-time fluorescence assays to show that engineered cathelicidins have a common mechanism of harming bacteria. A critical event is the induction of oxidative stress that damages the cells.

## RESULTS

### B22 and B22a kill multidrug-resistant enterobacteria.

**FIG 1:**
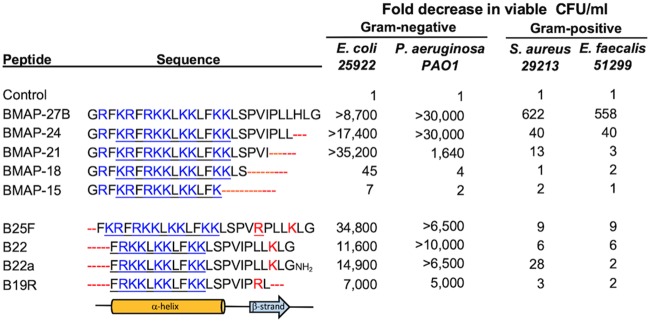
Antibacterial activity of BMAP-27B variants. (Left) The sequences of BMAP-27B and its truncated and charge variants are shown. The predicted secondary structures (α-helix and β-strand) of the cathelicidins are indicated below the sequences. (Right) The numbers represent the fold decrease in the CFU of bacterial cultures treated with 2 μM of each peptide against representative Gram-negative and Gram-positive bacteria.

We previously showed that truncating and increasing the overall positive charge of the bovine peptide BMAP-34 could reduce its activation of TLR3 signaling while increasing its bactericidal activity (10). These efforts led to our characterization of BMAP-27B, a derivative with potent bactericidal activity against Gram-negative bacteria. We sought to further engineer BMAP-27B to improve its bioactivities by shortening and changing specific residues. We enumerated viable Gram-negative (Escherichia coli and Pseudomonas aeruginosa) and Gram-positive (Staphylococcus aureus and Enterococcus faecalis) cells after 1 h of incubation with 2 μM of the peptide variants (Fig. 1). A peptide with a truncation of the three C-terminal residues (BMAP-24) was found to retain bactericidal activity against E. coli and P. aeruginosa, but it worsened its already poor activity against S. aureus and E. faecalis. Truncation of six C-terminal amino acids reduced killing of P. aeruginosa, without significantly affecting killing of E. coli. Longer C-terminal truncations caused a complete loss of bactericidal activity.

Truncations of one, two, or five N-terminal amino acids from BMAP-27B resulted in peptides that largely retained bactericidal activities against E. coli and P. aeruginosa, the shortest of which was B22. However, peptide B19R, which lacked five and three residues at the N and C termini, respectively, showed modestly reduced killing of E. coli, P. aeruginosa, and other Gram-negative bacteria. Thus, B22 and B22a (a C-terminal amide derivative anticipated to exhibit decreased exoprotease sensitivity) retained the potent bactericidal activity of BMAP-27B toward several Gram-negative bacteria without affecting activity toward Gram-positive bacteria and were selected for further testing as AMPs that can preferentially kill Gram-negative members of the family *Enterobacteriaceae*.

The MICs of BMAP-27B, B22, and B22a were determined against a panel of Gram-negative bacteria. B22 and B22a were as good or better than BMAP-27B at inhibiting the growth of Enterobacter cloacae, E. coli, Vibrio cholerae, Klebsiella pneumoniae, and P. aeruginosa (Table 1). Notably, V. cholerae that is naturally resistant to polymyxin and multidrug-resistant K. pneumoniae from regional health care facilities, including those carrying the New Delhi metallo-β-lactamase 1 (NDM1+) gene, were susceptible to BMAP-27B, B22, and B22a. Thus, truncated derivatives of BMAP-27B could inhibit the growth of multidrug-resistant members of the *Enterobacteriaceae*.

**TABLE 1:** MICs of B22 and B22a toward Gram-negative bacteria

Species and strain	MIC (μM)		
	BMAP-27B	B22	B22a
Enterobacter cloacae			
OC4080	4	4	4
OC4092	4	4	4

Escherichia coli			
ATCC 25922	4	2	2
ATCC 35218	4	4	2
IU342	2	4	2
J53 AzideR	4	4	2
OC4075	4	4	2
MC4100a	4	4	4
UTI89	4	2	2

Vibrio cholerae			
AC53	2	2	4
C6075	4	2	4

Klebsiella pneumoniae			
ATCC 700603	4	2	2
C2	4	4	2
OC4110	8	4	2
OC8893	8	4	2

88 (NDM1+)	4	4	2
262 (NDM1+)	4	4	4

Pseudomonas aeruginosa			
ATCC 27853	4	4	2
OC4083	4	2	2
PAO1	4	4	2
PAO1 oprD	4	4	2

### Reduced lysis of human red blood cells by B22 and B22a.

Mammalian cells are thought to be less susceptible to cathelicidins than bacteria in part because their membrane composition reduces interaction with the peptides. Hence, the lysis of human red blood cells (hRBCs) is a convenient and sensitive method to assess the detrimental effects of the peptides. We previously demonstrated that BMAP-27B exhibited decreased activation of innate immune responses in human cells and reduced hRBC lysis compared to LL-37 (10). However, cathelicidins of different lengths and sequences could differentially affect mammalian cell lysis. The effects of B22 and B22a on hemoglobin release from hRBCs were assessed after 1 h of incubation with each peptide. hRBC lysis in water (100%) was used as a reference. B22 and B22a were less toxic than LL-37 at 2 μM and showed minimal hemolysis relative to a phosphate-buffered saline (PBS control) (see Fig. S1A in the supplemental material). Moreover, LL-37 concentrations of >10 μM resulted in significant hRBC lysis, while concentrations of 15 μM or higher of B22 or B22a did not significantly increase hRBC lysis (Fig. S1B).

10.1128/mBio.02021-19.1 FIG S1 B22 and B22a are reduced for the lysis of human red blood cells. (A) Lysis of hRBCs by 2 mM concentration of the peptides. (B) Lysis of hRBCs by increasing concentrations of peptides. Download FIG S1, TIF file, 2.5 MB. Copyright © 2019 Rowe-Magnus et al. 2019 Rowe-Magnus et al. This content is distributed under the terms of the Creative Commons Attribution 4.0 International license.

### B22 permeabilizes the cytoplasmic membrane of V. cholerae.

To better understand the antimicrobial activity of the peptides, we developed a single-cell, real-time imaging assay to monitor the disruption of V. cholerae cells. Bacteria were seeded into microfluidic chambers and grown under continuous flow before imaging by phase-contrast microscopy. Untreated V. cholerae cells retained their characteristic comma shape, cell length (1 by 2 μm), and membrane integrity (Fig. S2). Conversely, cells treated with 2 μM B22 (or B22a) began losing their shape and were noticeably shorter within 30 s (Fig. S2). These cells did not recover even after continuous flow overnight in fresh medium, suggesting that they were no longer viable. Interestingly, the appearance of small circular bodies in the vicinity of compromised cells accompanied the loss in membrane integrity.

10.1128/mBio.02021-19.2 FIG S2 B22 induces membrane blebbing. Phase-contrast images of V. cholerae taken at 30 and 60 s after B22 treatment. (Top) Control sample to which no peptide was added. (Bottom) Sample treated with 2 μM B22. Red arrowheads indicate compromised cells. (Right) Dotted lines highlight the comma-shaped profile of control cells, and the misshapen profile of treated cells. Adjacent circular bodies (gray) are shown. Download FIG S2, TIF file, 1.0 MB. Copyright © 2019 Rowe-Magnus et al. 2019 Rowe-Magnus et al. This content is distributed under the terms of the Creative Commons Attribution 4.0 International license.

**FIG 2:**
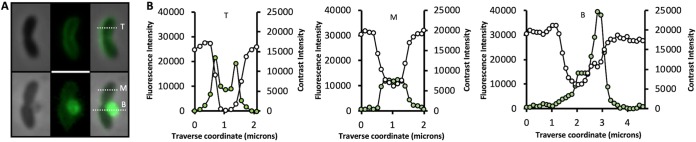
B22 permeabilizes the cytoplasmic membrane and induces blebbing. (A) Phase-contrast and fluorescent images of V. cholerae expressing periplasmic Tat^sf^GFP. (Top) A control cell to which no peptide was added. (Bottom) A cell 60 s after exposure to 2 μM B22. (B) Traverse signal intensity line scans for phase-contrast (white circles) and ^sf^GFP fluorescence (green circles) along the top (T), middle (M), and bottom (B) dotted white lines in panel A.

The change in shape observed for V. cholerae cells after peptide treatment could be due to permeabilization of the outer and/or cytoplasmic membranes. To determine whether either membrane was affected, we targeted superfolder green fluorescent protein (^sf^GFP) to the periplasmic space by fusing it to the N-terminal TorA twin-arginine translocase signal peptide (Tat^sf^GFP) (16). Control cells yielded high-contrast phase images (Fig. 2A, top) and traverse signal intensity line scans (Fig. 2B, left panel, white circles) indicative of intact peptidoglycan, chromosomal DNA, and larger cytoplasmic structures such as ribosomes. Fluorescence imaging of the same cells revealed a halo distribution for the ^sf^GFP signal in >90% of the cells and a bimodal transverse signal intensity pattern consistent with its periplasmic accumulation (Fig. 2B, left panel, green circles). In contrast, cells exposed to 2 μM B22 exhibited reduced contrast (Fig. 2B, middle panel, white circles), and the fluorescent signal was uniformly distributed in treated cells, indicating rapid translocation of ^sf^GFP from the periplasm to the cytoplasm (Fig. 2B, middle panel, green circles). These results suggested that B22 permeabilized the cytoplasmic membrane while leaving the outer membrane intact, since the latter would have resulted in the loss of ^sf^GFP signal. The GFP signal was eventually concentrated within an expanding membrane bleb that originated from the compromised cell (Fig. 2B, right panel, green circles).

**FIG 3:**
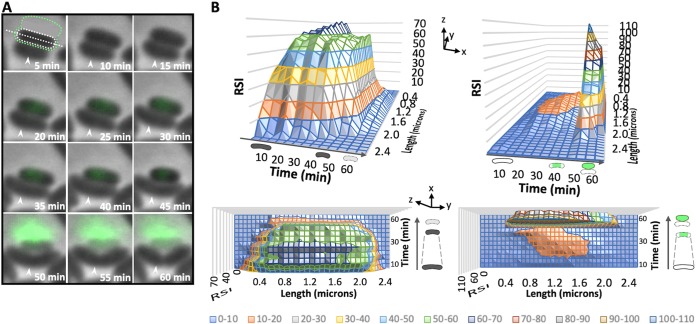
Loss of membrane integrity, cell shortening, and expulsion of DNA after B22 treatment. (A) Time-lapse phase-contrast and fluorescent image overlays on agarose pads of V. cholerae cells following treatment with 1 μM B22. SYTOX Green was present in the medium at a final concentration of 2.5 μM. SYTOX Green is impermeant to live cells, and only dead or dying cells fluoresce. The cell length (white dotted line) and membrane integrity measured as the relative signal intensity (RSI) by phase-contrast microscopy (dotted white region) and SYTOX Green fluorescence (dotted green region) were determined in each image for dying cells. The white arrowheads indicate an actively dividing cell. (B) Surface plots quantifying the change in cell length/membrane integrity (left) and fluorescence (right) of the dying cell in panel A. Shown are the side (above) and top-down (below) views of the decrease in cell length (*z* axis) and change in membrane integrity or fluorescence (*y* axis) over time (*x* axis) as the cell dies. A schematic representation of events (changes in cell contrast, cell length, and GFP signal) is shown above and beside each plot. Dashed lines highlight the cell shortening event. The relative signal intensity color key is shown at the bottom of the figure.

To better understand the membrane blebbing event, V. cholerae was treated with B22 in the presence of SYTOX Green and imaged on agarose pads to stabilize cells with compromised envelopes. SYTOX Green is a fluorescent nucleic acid stain that is impermeant to live cells and is used to distinguish dead or dying cells in a population (17). Cells were treated with a sublethal (1 μM) concentration of B22 in order to visualize physiological changes in susceptible and unaffected cells simultaneously. Time-lapse phase-contrast imaging was used to monitor tip-to-tip cell length and membrane integrity. Unaffected V. cholerae cells exhibited little SYTOX Green staining and retained their characteristic comma shape, cell length, and membrane integrity, and cells continued to divide (Fig. 3A, white arrowheads). A strong cytoplasmic SYTOX Green signal was observed in approximately 35% of the population (Fig. 3A and B). These dying cells exhibited an 10 to 20% reduction in cell length that likely resulted from leakage of osmolytes across the cell membrane and the subsequent loss of turgor pressure. A sudden and nearly complete loss in cell contrast (>90% decrease) coincided with an abrupt burst of fluorescence exterior to the dying cell, suggesting that the DNA was expelled. The loss in cell contrast, the accumulation of cytoplasmic B22^FAM^ signal in membrane blebs and SYTOX Green-DNA signal exterior to the cell collectively suggested that the cytoplasmic contents of the cell were displaced into the blebs.

**FIG 4:**
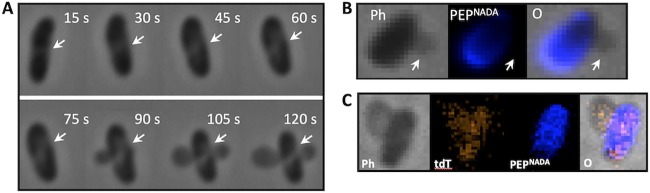
B22 enters the cytoplasm and induces membrane blebbing in regions of active peptidoglycan turnover. (A) Time-lapse phase-contrast and fluorescent images of V. cholerae cells treated with 2 μM B22. White arrows indicate regions of reduced optical density. (B) V. cholerae cells were grown in the presence of NADA (pseudocolored blue) for three generations to thoroughly label the peptidoglycan, washed, and then chased in fresh medium containing 2 μM B22. Images were taken 2 min later. A representative cell is shown with phase-contrast (Ph), labeled peptidoglycan (PEP^NADA^), and overlay (O) images. White arrows indicate points of membrane blebbing. (C) V. cholerae cells expressing tdTomato (red labeling) were grown in the presence of NADA (blue labeling) for three generations to thoroughly label the peptidoglycan. Phase-contrast (Ph), tdTomato (tdT), labeled peptidoglycan (PEP^NADA^), and overlay (O) images are shown.

To investigate cytoplasmic extrusion in greater detail, we captured time-lapse phase-contrast images of V. cholerae cells treated with B22. Zones of low contrast were apparent within 30 s of peptide addition (Fig. 4A). Shortly thereafter, blebs were observed originating from these zones. We hypothesized that the low-contrast zones corresponded to regions of active peptidoglycan turnover. To examine this, V. cholerae was first grown in the presence of NADA (18) for three generations to completely label the peptidoglycan (PEP^NADA^). The cells were then washed and resuspended in fresh medium containing 2 μM B22. Membrane blebs protruding from treated cells were readily observed (Fig. 4B). Notably, fluorescence imaging of the same cells revealed that bleb formation coincided with regions of active peptidoglycan turnover (dark areas of the cell in which the labeled peptides have been replaced). Moreover, the blebs contained both 4′,6′-diamidino-2-phenylindole (DAPI)-labeled DNA and fluorescent tdTomato that was expressed in the cytoplasm (Fig. 4C). Collectively, these results suggested that B22 led to a weakening of the peptidoglycan in regions of active turnover and extrusion of the cytoplasmic contents into membrane-bound blebs that originated within these regions. Moreover, both entities remained stable after 24 h, suggesting that the bactericidal effects of the peptides did not discharge endotoxin or peptidoglycan into the medium. Similar results were obtained with B22a. These data showed that the treatment of V. cholerae with micromolar concentrations of B22 or B22a resulted in the formation of an empty sacculus remnant and external membrane-bound blebs that contained displaced cytoplasmic material.

### B22 enters the cytoplasm of Gram-positive bacteria.

**FIG 5:**
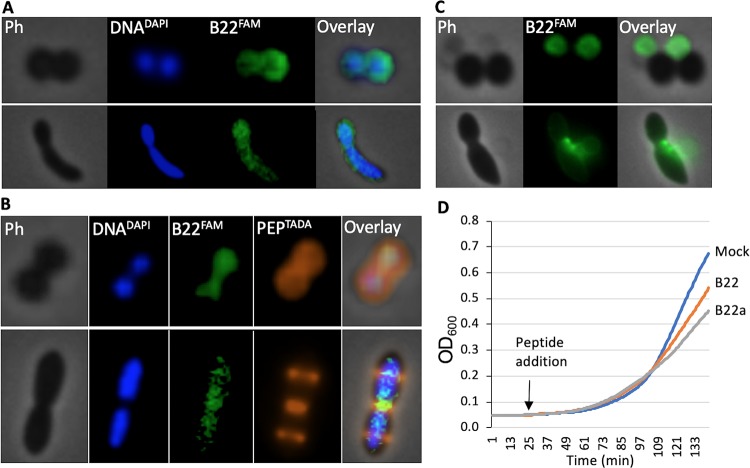
B22 rapidly binds to Gram-positive bacteria, enters the cytoplasm, and slows growth. (A and C) Phase-contrast (Ph) and fluorescent imaging of S. aureus (top row) and E. faecalis (bottom row) in which their DNA was labeled with DAPI (DNA^DAPI^) followed by treatment with 5 μM FAM-labeled B22 (B22^FAM^). In panel A, the cells were imaged after 1 min. In panel C, the cells were imaged after 60 min. (B) Imaging in which the peptidoglycan was also labeled with TADA (PEP^TADA^). The cells were imaged after 30 min. (D) Growth of S. aureus after the addition of 30 nM B22 peptide (orange line) or B22a peptide (gray line) or PBS was added (Mock [blue line]).

Much higher concentrations of B22 and B22a were needed to kill Gram-positive bacteria (Fig. 1). Since cathelicidins are also released in response to infection by Gram-positive bacteria (19), we sought to determine why B22 did not kill Gram-positive cells at concentrations that are highly effective against Gram-negative cells. 6-Carboxyfluorescein (FAM)-labeled B22 (B22^FAM^), a fluorescein-labeled peptide that has MICs for Gram-negative bacteria comparable to those of B22, bound to S. aureus and E. faecalis within 1 min of exposure (Fig. 5A), demonstrating that the peptide could penetrate the Gram-positive cell wall. To better visualize cellular structures, we labeled the peptidoglycan with the fluorescent probe tetramethylrhodamine 3-amino-d-alanine (PEP^TADA^) and DNA with DAPI (DNA^DAPI^), and localized B22 distribution. For both S. aureus and E. faecalis, the phase-contrast and PEP^TADA^ images overlapped entirely and delineated the outer cell boundary, while the DNA^DAPI^ signal was confined to the interior of the cell (Fig. 5B). The B22^FAM^ signal was readily localized to the cytoplasm in both species. Extended incubation times (60 min) resulted in concentration of the B22^FAM^ signal and the formation of membrane blebs similar to those observed with V. cholerae (Fig. 5C). Thus, B22 was able to quickly enter the cell cytoplasm of Gram-positive cells despite the fact that it exhibited minimal bactericidal activity against these cells.

While the numbers of viable S. aureus and E. faecalis colonies did not change after treatment with low micromolar concentrations of B22 or B22a (Table 1), the colonies were smaller in diameter compared to untreated samples. These observations suggested that B22 affected functions needed for growth, even at concentrations lower than the MIC. To examine this further, we monitored the growth of S. aureus cultures after the addition of 30 nM B22 or B22a. This concentration is in the biologically relevant range of peptide that is produced by mammals (13). Notably, sublethal levels of B22 or B22a reduced the initial growth rate of S. aureus (Fig. 5D). Cell growth recovered at later time points (data not shown). These results suggested that sub-MIC levels of cathelicidins can negatively impact processes important for bacterial growth.

### Sublethal concentrations of B22 slow Gram-negative bacterial growth.

**FIG 6:**
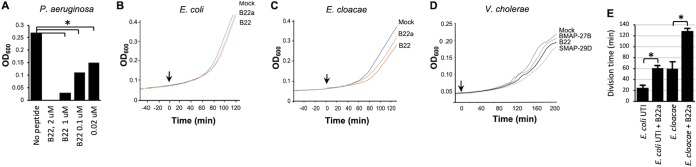
Cathelicidins slow the growth of Gram-negative bacteria at nanomolar concentrations. (A to D) Growth of P. aeruginosa in increasing concentrations of B22 (A) and E. coli, E. cloacae, and V. cholerae in the presence of 30 nM concentration of the indicated peptide (B to D). (E) E. coli and E. cloacae were incubated without B22a or with 30 nM B22a. Cell division time was recorded by time-lapse phase-contrast microscopy. The lines in panels B to D represent the means for four independent data sets. Control cells to which PBS was added (Mock) are shown. The bar plots in panels A and E show the means, and error bars represent the standard deviations. Values that are significantly different (*P* < 0.001) are indicated by a bar and asterisk.

In light of the effect of sublethal concentrations of cathelicidins on the growth of Gram-positive bacteria, we tested the impact of sublethal concentrations of B22 on the growth rate of Gram-negative bacteria. Relative to the no-peptide control, a pronounced decrease in the growth of P. aeruginosa was observed after treatment with sublethal doses of B22 for 2 h (Fig. 6A). Even at 20 nM B22, the initial growth of P. aeruginosa was significantly reduced relative to the control. Likewise, nanomolar concentrations of B22 or B22a reproducibly reduced the growth rate of E. coli, E. cloacae, and V. cholerae (Fig. 6B to D). Furthermore, these results were not restricted to engineered cathelicidins, as BMAP-27B and SMAP29D all had a similar inhibitory effect on the growth of V. cholerae (Fig. 6D).

To confirm that sublethal levels of cathelicidins affected the growth of Gram-negative bacteria, the division of single cells of E. coli and E. cloacae was monitored. Cells were seeded into microfluidic chambers and grown under continuous flow, and time-lapse phase-contrast microscopy was used to record the cell division time in the presence or absence of B22a. Although the bacterial cells continued to grow and divide, the division time for both E. coli and E. cloacae increased twofold over the first three generations in the presence of 30 nM B22a (Fig. 6E). These results suggested that sublethal concentrations of cathelicidins negatively impacted cell division of Gram-negative bacteria.

### B22 affects bacterial gene expression.

To determine the mechanism by which sublethal concentrations of cathelicidins affect bacterial cell physiology, we sought to analyze gene expression prior to and after a 5-min exposure to 30 nM B22a. The chromosomal integron of V. cholerae contains hundreds of repeats and horizontally acquired genes of unknown function (20–23). Moreover, many of these genes are duplicated, which can further complicate interpreting gene expression patterns. We elected to examine peptide-induced expression changes in E. coli to simplify gene identification and assignment of potential functions. Significant changes in expression were identified for genes whose products participate in global processes such as protein synthesis, protein folding, protein secretion, intracellular pH, membrane modification, c-di-GMP production, peptidoglycan biosynthesis, respiration, and detoxification of reactive oxygen species (ROS) (Table 2). Perturbation of peptidoglycan synthesis was consistent with the membrane blebbing we observed following B22 and B22a treatment. Other notable changes included *arnF*, the product of which modifies the lipid A moiety of lipopolysaccharide (LPS) with 4-amino-4-deoxy-l-arabinose (l-Ara4N) to confer resistance to polymyxin and cationic antimicrobial peptides in E. coli and Salmonella enterica serotype Typhimurium (24, 25). c-di-GMP-dependent Psl exopolysaccharide production is part of the first line of defense against reactive oxygen species in P. aeruginosa (26). The respiratory chain is a major source of periplasmic ROS such as superoxide (O_2_^−^) and hydrogen peroxide (H_2_O_2_) (27), and cytosolic flavoenzymes such as fumarate reductase (Frd) can generate O_2_^−^, H_2_O_2_, and hydroxyl radical (^·^OH) that damages membrane lipids, DNA, and proteins. ArcA is important for regulating bacterial resistance to ROS under aerobic conditions, and OxyR directly senses H_2_O_2_ to induce expression of a slew of genes, including *ahpC* and *gshA*, to counter H_2_O_2_ accumulation (28, 29). The broad spectrum of differentially expressed genes suggested that B22a had a general toxic effect on bacteria that could simultaneously impact multiple cellular processes.

**TABLE 2:** Differentially expressed E. coli UTI89 genes after treatment with 30 nM B22a

Change and gene ID	Log_2_ fold change	*P* value	KEGG function	Gene
Increased expression				
UTI89_C1760	4.38	8.39E−53	c-di-GMP diguanylate cyclase	*ydeH*
UTI89_C1669	2.96	4.39E−09	DNA-binding transcriptional regulator	*mcbR*
UTI89_C2044	2.85	4.39E−09	DNA polymerase III subunit theta	*holE*
UTI89_C1304	2.56	1.02E−26	Prophage endopeptidase	*arrD*
UTI89_C4848	2.24	1.57E−14	Enamine/imine deaminase	*yjgF* (*ridA*)
UTI89_C1303	2.14	2.39E−05	Prophage lysozyme	*ybcS1*
UTI89_C2239	1.92	2.20E−03	Hypothetical protein	*yaiS*
UTI89_C3644	1.90	8.86E−05	Peptidoglycan glycosyltransferase	*mtgA*
UTI89_C0124	1.89	1.30E−06	Hypothetical protein	*orf*
UTI89_C0165	1.85	2.82E−12	Peptidoglycan synthase	*mrcB*
UTI89_C3670	1.80	2.91E−04	Ribonuclease inhibitor	*yhcO*
UTI89_C0453	1.80	1.57E−13	Hypothetical protein	*cyoD*
UTI89_C1348	1.77	1.51E−03	Hypothetical protein	*ymgA*
UTI89_C4048	1.74	1.30E−03	Transcriptional regulator	*gadX*
UTI89_C1051	1.67	2.70E−04	Cold shock protein	*cspG*
UTI89_C1172	1.63	1.36E−03	Periplasmic glucan biosynthesis protein	*mdoG*
UTI89_C0338	1.57	1.17E−03	Type 1 fimbria regulatory protein	*fimX*
UTI89_C1095	1.47	8.77E−04	Hypothetical protein	*orf*
UTI89_C4097	1.44	3.98E−04	Cold shock protein	*cspA*
UTI89_C2541	1.44	1.21E−08	Undecaprenyl phosphate-l-ara4N flippase subunit	*arnF* (*yfbJ*)
UTI89_C1152	1.40	9.36E−10	Hypothetical protein	*orf*
UTI89_C0130	1.39	2.93E−05	Hypothetical protein	*yacH*
UTI89_C1870	1.34	5.84E−05	Peptidoglycan d-transpeptidase	*ynhG*
UTI89_C0026	1.32	4.08E−09	Hypothetical protein	*yaaY*
UTI89_C3182	1.31	4.40E−09	Hypothetical protein	*ygdI*
UTI89_C0880	1.31	5.86E−07	ATP-dependent endonuclease	*ybjD*
UTI89_C3181	1.30	1.36E−04	Hypothetical protein	*ygdI*
UTI89_C2702	1.30	1.83E−03	Two-component system acid-sensing sensor kinase	*evgS*
UTI89_C2303	1.29	1.31E−03	Hypothetical protein	*wbgM*
UTI89_C1833	1.29	7.20E−08	Transcriptional regulator of multiple antibiotic resistance	*marR*
UTI89_C1604	1.28	5.63E−06	Universal stress protein E	*uspE*
UTI89_C1899	1.27	5.87E−07	Hypothetical protein	*ydiU*
UTI89_C0686	1.22	2.49E−05	Hypothetical protein	*ybfN*
UTI89_C0505	1.20	4.83E−03	Inosine kinase	*gsk*
UTI89_C4481	1.13	3.04E−06	Hypothetical protein	*fdoG*
UTI89_C3236	1.12	3.41E−05	Hypothetical protein	*ygdR*
UTI89_C3330	1.11	6.56E−07	Hypothetical protein	*yqgD*
UTI89_C0566	1.11	4.49E−03	Outer membrane protein	*ompT*
UTI89_C4554	1.11	2.19E−03	Hydrogen peroxide-inducible transcriptional activator	*oxyR*
UTI89_C4616	1.10	2.83E−04	Fur family transcriptional regulator	*yjbK*
UTI89_C3928	1.10	3.73E−03	Hypothetical protein	*orf*
UTI89_C3736	1.09	1.95E−04	Ribosome rescue factor	*yhdL*
UTI89_C1286	1.09	4.26E−03	Hypothetical protein	*orf*
UTI89_C4521	1.08	1.74E−04	50S ribosomal subunit protein L31	*rpmE*
UTI89_C1169	1.04	4.75E−03	Cardiolipin synthase	*ymdC*
UTI89_C2077	1.03	8.93E−04	Cytochrome *c* trimethylamine-*N*-oxide reductase	*torY*
UTI89_C3724	1.02	2.07E−05	Hypothetical protein	*yrdA*

Decreased expression				
UTI89_C2748	−1.06	1.64E−03	Hypothetical protein	*ptsK*
UTI89_C2386	−1.09	1.18E−03	Hypothetical protein	*yehE*
UTI89_C2497	−1.11	5.14E−03	Outer membrane pore protein C	*ompC*
UTI89_C3587	−1.14	1.92E−03	Hypothetical protein	*yhbW*
UTI89_C1622	−1.15	8.31E−05	Heat shock protein HslJ	*hslJ*
UTI89_C2016	−1.16	1.97E−06	Mannose-specific enzyme IIC component of PTS	*manY*
UTI89_C4503	−1.16	1.76E−03	Triosephosphate isomerase	*tpiA*
UTI89_C1002	−1.19	2.19E−03	Asparaginyl-tRNA synthetase	*asnS*
UTI89_C2472	−1.19	4.17E−03	Cytochrome *c*-type biogenesis protein CcmH	*ccmH*
UTI89_C3842	−1.22	1.78E−03	Elongation factor G	*fusA*
UTI89_C4647	−1.25	3.42E−05	c-di-GMP phosphodiesterase	*yjcC*
UTI89_C1537	−1.27	2.04E−03	23S rRNA pseudouridine synthase	*yciL*
UTI89_C4739	−1.27	1.33E−04	Amino acid efflux transporter	*yjeH*
UTI89_C0608	−1.35	2.27E−03	Peroxiredoxin hydroperoxide reductase subunit C	*ahpC*
UTI89_C3746	−1.36	1.49E−04	Preprotein translocase subunit	*secY*
UTI89_C4299	−1.38	2.48E−03	Aspartate-ammonia ligase	*asnA*
UTI89_C2011	−1.39	5.23E−03	c-di-GMP phosphodiesterase	*ardB*
UTI89_C2753	−1.41	4.31E−03	Hypothetical protein	*yfeK*
UTI89_C2302	−1.41	1.03E−03	6-Phosphogluconate dehydrogenase	*gnd*
UTI89_C4380	−1.48	2.31E−03	Hypothetical protein	*orf*
UTI89_C1019	−1.48	2.79E−03	3-Hydroxyacyl-[acyl-carrier protein] dehydratase	*fabA*
UTI89_C5174	−1.55	1.24E−03	Two-component system response regulator	*arcA*
UTI89_C2387	−1.64	2.58E−03	ATP-binding protein involved in chromosome partitioning	*mrp*
UTI89_C0972	−1.66	3.04E−06	Major facilitator superfamily transporter	*ycaD*
UTI89_C3582	−1.70	7.37E−07	Endonuclease	*yhbQ*
UTI89_C4612	−1.71	5.00E−05	Diacylglycerol kinase	*dgkA*
UTI89_C4742	−1.76	2.36E−03	Hypothetical protein	*yjeI*
UTI89_C4754	−1.78	5.48E−04	Fumarate reductase	*frdA*
UTI89_C1779	−1.82	1.21E−09	Chloride channel protein	*ynfJ*
UTI89_C2583	−1.88	4.06E−05	NUDIX hydrolase	*yfcD*
UTI89_C2573	−1.89	2.63E−05	5'-Deoxynucleotidase	*yfbR*
UTI89_C3050	−1.92	8.00E−07	Glutaredoxin	*gshA*
UTI89_C3862	−1.97	7.31E−04	*N*-Succinyldiaminopimelate aminotransferase	*dapC*
UTI89_C2471	−2.02	1.39E−09	Two-component system response regulator	*narP*
UTI89_C3148	−2.02	4.78E−07	Enolase	*eno*
UTI89_C0216	−2.02	4.12E−03	d,d-Heptose 1,7-bisphosphate phosphatase	*gmhB*
UTI89_C3686	−2.10	6.18E−07	Hypothetical protein	*yrdE*
UTI89_C3411	−2.15	9.56E−10	Disulfide bond oxidoreductase and hydroperoxidase	*yghU*
UTI89_C3167	−2.21	2.41E−05	Serine transporter	*sdaC*
UTI89_C2105	−2.29	3.03E−03	Hypothetical protein	*yecR*
UTI89_C0908	−2.29	3.74E−04	Seryl-tRNA synthetase	*serS*
UTI89_C4736	−2.31	1.94E−13	Aspartate ammonia-lyase	*aspA*
UTI89_C2517	−2.34	4.73E−07	Ferredoxin	*yfaE*
UTI89_C1975	−2.34	8.86E−08	Glyceraldehyde 3-phosphate dehydrogenase	*gapA*
UTI89_C3253	−2.37	2.08E−03	Xanthine dehydrogenase	*xdhC*
UTI89_C4151	−2.45	1.22E−03	Protein export protein	*secB*
UTI89_C2480	−2.52	8.86E−04	Cytochrome *c*-type protein NapC	*napC*
UTI89_C2754	−2.60	4.23E−04	Cysteine synthase	*cysM*
UTI89_C4735	−2.63	9.23E−04	Anaerobic C4-dicarboxylate transporter	*dcuA*
UTI89_C0624	−2.71	1.45E−08	Two-component response regulator	*citB*
UTI89_C0974	−2.71	6.40E−12	Formate acetyltransferase	*pflB*
UTI89_C4840	−2.73	8.22E−10	Soluble cytochrome *b*562	*cybC*
UTI89_C3920	−2.90	1.14E−05	RNA 3'-terminal phosphate cyclase	*rtcA*
UTI89_C0333	−2.93	1.27E−07	Inner membrane protein	*ykgH*
UTI89_C4753	−2.97	1.06E−04	Fumarate reductase	*frdB*
UTI89_C3168	−3.16	4.37E−04	l-Serine dehydratase	*sdaB*
UTI89_C3346	−3.26	7.14E−13	l-Asparaginase	*ansB*

### B22a triggers oxidative stress in V. cholerae.

**FIG 7:**
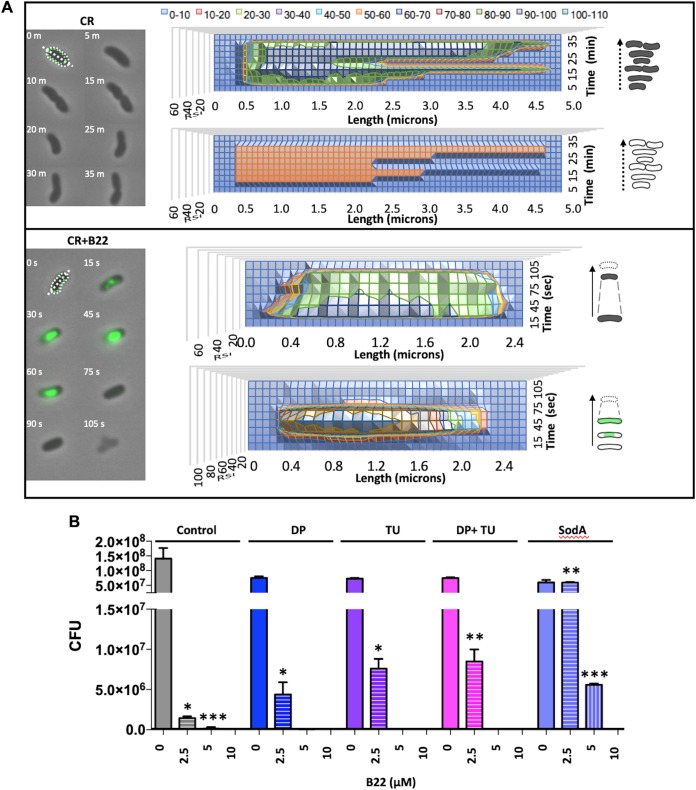
B22 induces oxidative stress, cell shortening, and membrane blebbing. (A) Time-lapse phase-contrast and fluorescent image overlays of V. cholerae after exposure to B22. (Top) Control cells in medium with CellROX (CR); (bottom) cells in medium with CR and 100 nM B22 (CR+B22). The top graph in each panel displays the change in cell length and optical density (contrast) over time. The bottom graph shows the relative fluorescence signal intensity (RSI) of cells, which increases with the accumulation of reactive oxygen species. To the right are schematics of the events quantified in each graph. Dashed lines highlight the cell shortening event. (B) Sensitivity (CFU) of V. cholerae cells to increasing concentrations of B22 in the presence or absence of 75 μM dipyridyl (DP), 150 mM thiourea (TU) alone or in combination or with expression of the superoxide dismutase, SodA. Control cells were treated with PBS. Values for treated cells and the respective control cells that exhibit statistically significant differences are indicated by asterisks as follows: *, *P* < 0.01; **, *P* < 0.001; ***, *P* < 0.0001.

The assortment of E. coli genes that were differentially expressed after peptide treatment led us to suspect that B22a induced oxidative stress. Oxidative stress is often transient and challenging to detect due to a suite of enzymes in bacterial cells that can rapidly reduce oxidative species. We monitored the real-time accumulation of ROS in single cells of V. cholerae in the presence of CellROX, a cell-permeable redox dye that reacts with O_2_^–^ and ^·^OH (30). CellROX is nonfluorescent in the reduced state, but upon oxidation, it binds double-stranded DNA and fluoresces strongly. Images were captured at 15-s intervals over 40 min, twice the doubling time of V. cholerae under these conditions. No change in membrane integrity or CellROX fluorescence of control cells was detected over the first 2 min (Fig. S3) or at any time during the first division round (Fig. 7A, top), indicating that the normal O_2_^–^ levels and free cellular Fe^2+^ concentration were too low to support ^·^OH formation (31). The number of fluorescent cells increased following treatment with 25, 50, or 100 nM B22 or B22a (Fig. S4), and a fluorescence burst due to the oxidation of CellROX was observed within 15 s of B22 treatment (Fig. 7A, bottom). The signal intensity peaked over the next 30 s and then dissipated after about 1 min. The loss in fluorescence intensity was followed by an abrupt shortening in cell length, a dramatic decrease in cell contrast, and bleb formation roughly 45 s later. Collectively, these results suggested that bacterial membrane blebbing following B22 treatment is preceded by the accumulation of ROS that affected global cellular processes, including peptidoglycan biosynthesis.

10.1128/mBio.02021-19.3 FIG S3 B22 induces oxidative stress, cell shortening, and membrane blebbing. Time-lapse phase-contrast and fluorescent image overlays of V. cholerae control cells in medium with CellROX (CR). Images were captured every 15 s. The cell length (dotted white line), contrast (dotted white region), and relative fluorescence signal intensity (dotted green region) were measured over time. Download FIG S3, TIF file, 0.3 MB. Copyright © 2019 Rowe-Magnus et al. 2019 Rowe-Magnus et al. This content is distributed under the terms of the Creative Commons Attribution 4.0 International license.

10.1128/mBio.02021-19.4 FIG S4 B22 induces membrane damage at nanomolar concentrations. V. cholerae was treated with increasing concentrations of B22 in the presence of SYTOX Green, and fluorescence was measured. Plots show the mean of the relative fluorescence intensity, and error bars represent the standard deviations. *P* values were calculated using the Student *t* test. Statistical significance is indicated as follows: *, *P* < 0.001. Download FIG S4, TIF file, 0.7 MB. Copyright © 2019 Rowe-Magnus et al. 2019 Rowe-Magnus et al. This content is distributed under the terms of the Creative Commons Attribution 4.0 International license.

### ROS scavengers and superoxide dismutase suppress B22-mediated cell death.

If the generation of ROS contributed to the physiological changes in Gram-negative bacteria, then agents and enzymes that reduce O_2_^–^ accumulation or downstream Fenton-driven ^·^OH formation should increase cell survival. The membrane-permeable iron chelating agent 2,2′-dipyridyl (DP) efficiently chelates free Fe^2+^ and the scavenger thiourea (TU) directly quenches hydroxyl radicals generated by the Fenton reaction. Treatment of V. cholerae with 2.5 μM B22 decreased survival by 3 log_10_ units (Fig. 7B). Preincubation with 75 μM DP or 150 mM TU alone or in combination prior to the addition of B22 increased V. cholerae survival by 7- and 13-fold, respectively. Increased expression of superoxide dismutase (SOD), which converts O_2_^–^ to H_2_O_2_, would also be anticipated to increase cell survival. Indeed, overproduction of SodA in V. cholerae completely suppressed killing by 2.5 μM B22. In the presence of 5 μM B22, V. cholerae overexpressing SodA exhibited only a 10-fold decrease in survival. These results demonstrate that O_2_^–^ and ^·^OH accumulation play critical roles in the killing of V. cholerae by B22.

### Cathelicidins induce ROS production in Gram-positive bacteria.

**FIG 8:**
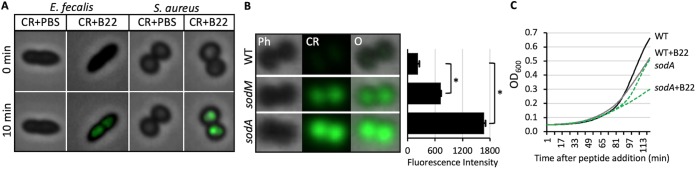
Peptide treatment induces ROS production and slows growth in wild-type S. aureus and *sod* mutants. (A) Overlay of phase-contrast and fluorescent images of E. faecalis and S. aureus at 0 and 10 min after treatment with PBS or 500 nM B22 in medium with 2.5 μM CellROX (CR). (B) Phase-contrast (Ph), CellRox (CR), and overlay (O) images of wild-type S. aureus (WT), *sodA*, and *sodM* cells that were treated with 100 nM B22 for 30 min in the presence of 2.5 μM CellRox. Quantification of fluorescence is shown to the right. The bar graph shows the mean fluorescence intensity values, and error bars represent the standard deviations. Measurements were made on 100 single cells per strain, and experiments were done in triplicate. *P values* were calculated using the Student’s *t* test. Significance: *, *P* < 0.001. (C) Growth of wild-type S. aureus and *sodA* cells in the presence or absence of 30 nM B22. Lines represent the means for four independent data sets. PBS was added to control cells.

We hypothesized that the decreased growth rate of Gram-positive bacteria in the presence of sublethal levels of cathelicidins was also due to the production of reactive oxygen species, and a fluorescent burst was observed when S. aureus and E. faecalis were treated with 500 nM B22 in the presence of CellROX (Fig. 8A). Furthermore, a strong oxidative signal was detected when S. aureus *sodA* or *sodM* null mutants were treated with 100 nM B22 or B22a, while only a weak signal was detected for the parental strain (Fig. 8B). The growth rate of the *sodA* mutant in the absence of peptide was lower than that of the wild type (WT), underscoring the importance of effectively controlling O_2_^−^ accumulation. The addition of B22 slowed the growth of the WT strain and, to an even greater extent, the *sodA* mutant (Fig. 8C). These results demonstrated that oxidative stress induced by cathelicidins plays a role in suppressing Gram-positive bacterial growth and that cytochrome oxidases help counter these detrimental effects.

### Cathelicidin oligomerization is not required for the growth inhibitory effects of B22.

**FIG 9:**
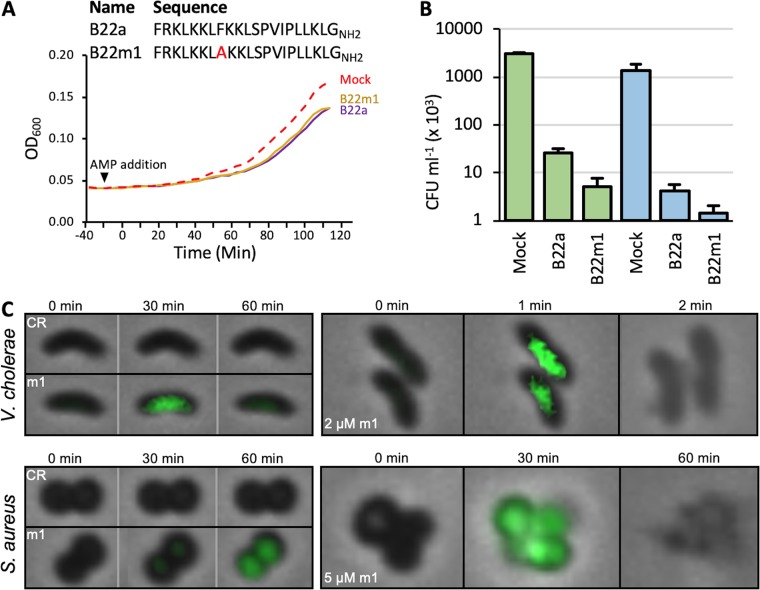
Cathelicidin oligomerization is not required for the growth inhibitory effects of B22. (A) The sequences of B22a and the variant B22m1 are shown at the top. A graph of the growth of P. aeruginosa PAO1 in the presence of 30 nM B22a or B22m1 or absence (mock) of B22a or B22m1 is shown below. (B) Viable colony counts of K. pneumoniae strain 92 and E. coli OC4075 after 1-h treatment in the absence (mock) or presence of 2 μM B22a or B22m1. (C, left) Time-lapse overlay of phase-contrast and fluorescent images of V. cholerae and S. aureus in medium containing 2.5 μM CellRox (CR) without or with 30 nM B22m1 (m1). (Right) Imaging of V. cholerae and S. aureus cells following treatment with 2 or 5 μM B22m1, respectively.

Cathelicidins are proposed to form higher-order structures in the bacterial membrane via intermolecular interactions between residues in the hydrophobic faces of their amphipathic helices. The observation that sublethal concentrations of B22 or B22a could induce oxidative stress and inhibit growth of both Gram-positive and Gram-negative bacteria led us to speculate that the formation of higher-order structures by B22 was not required for its bacteriostatic/cidal activity. To assess this, a variant that harbors a phenylalanine-to-alanine substitution at residue 8 within the amphipathic α-helix (B22m1) was generated. This position is predicted to mediate cathelicidin oligomerization (Fig. S5A). Dynamic light scattering spectroscopy showed that B22m1 formed structures that were much smaller than those formed by B22 and B22a (Fig. S5B). Sublethal concentrations of B22m1 (30 nM) reduced the growth rate of P. aeruginosa to the same extent as B22a (Fig. 9A) and was even more effective than B22a at killing *K. pneumonia* and E. coli (Fig. 9B). B22m1 also induced ROS production in both V. cholerae and S. aureus at sublethal concentrations and caused cell lysis at micromolar concentrations (Fig. 9C). These results suggested that oligomerization between cathelicidin monomers is not required for toxicity.

10.1128/mBio.02021-19.5 FIG S5 Mutant peptide B22m1 is defective in forming higher-order structures *in vitro*. (A) A model for the interaction between hydrophobic residues in B22. The helical wheel of the amphipathic α-helices of four B22 molecules are shown. The positively charged residues are colored blue and the hydrophobic residues are colored yellow. Phenylalanine 8 is outlined in green. The N- and C-terminal residues of each helix are indicated by n and c, respectively. (B) Hydrodynamic diameters (in decinanometers [dnm]) for the cathelicidin peptides B22, B22a, and B22m1. All three peptides were at a final concentration of 100 mM in phosphate-buffered saline (pH 7.4) at 21°C. The data were reproducible for six independent scans, although the mean diameters varied by ca. 20%. The data were collected using a Zetasizer dynamic light scatter spectrometer (Malvern Instruments). Download FIG S5, TIF file, 2.4 MB. Copyright © 2019 Rowe-Magnus et al. 2019 Rowe-Magnus et al. This content is distributed under the terms of the Creative Commons Attribution 4.0 International license.

### B22 can penetrate and kill established V. cholerae and P. aeruginosa biofilms.

**FIG 10:**
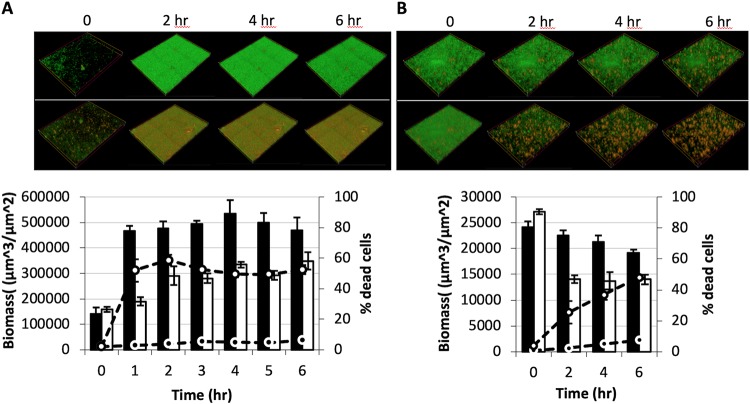
Effect of B22 on established P. aeruginosa and V. cholerae biofilms. (A and B) LIVE/DEAD (green/red) staining at 2-h intervals of P. aeruginosa (A) and V. cholerae (B) biofilms not treated with B22 (top panel) and after treatment with 100 nM B22 (bottom panel). Quantification of the total biomass for untreated (black bars) and treated (white bars) biofilms is shown in the bar graphs. The percentages of dead cells within the biofilms (untreated, black circles; treated, white circles) are shown.

B22 could traverse a myriad of complex cell surface structures: the acetylated LPS of colistin-resistant bacteria, the amino acid-modified LPS of a V. cholerae El-Tor isolate, the capsular polysaccharide (CPS) of Vibrio vulnificus, and the thick Gram-positive cell wall. These observations lead us to reason that B22 could penetrate and kill bacterial biofilms. P. aeruginosa was seeded into separate microfluidic flow cell chambers, and biofilm development and cell viability were tracked hourly under continuous flow. The biofilm development profile for control cells not exposed to B22 indicated a stably maintained biomass (black bars) and low death rate (black circles) over the course of the assay (Fig. 10A). Conversely, treatment with 100 nM B22 caused a 50% drop in biomass (white bars) over the same 4-h period. Moreover, a spike in cell death (>50 [white circles]) was detected after 1 h and remained high after 4 h. Similar effects were observed against V. cholerae biofilms. Again, control cells stably maintained biomass, cell death was minimal, and biofilm development was robust. In contrast, B22a treatment induced cell death (50% at 6 h) and decreased the overall biomass by half (Fig. 10B). These results suggested that B22 was effective at killing established P. aeruginosa and V. cholerae biofilms.

## DISCUSSION

Virtually all living organisms produce peptides that participate in defense against pathogens (32). One class of AMPs produced by metazoans are the cathelicidins, which are known to interact with and disrupt anionic bacterial lipid bilayers (33, 34). To design improved cathelicidins, it is important to understand how they function and their mechanism of action. In this work, we used high-resolution, single-cell, time-lapse imaging to reveal the timing, spatial distribution, and sequence of events underlying the bactericidal effects of engineered cathelicidins on bacterial cells. We found that an early effect of cathelicidins on bacterial cells was the induction of oxidative stress, even at nanomolar concentrations, that resulted in decreased bacterial growth. At higher peptide concentrations, this stress could initiate the collapse of cellular structures that led to bacterial death and did not require normal interaction between cathelicidin peptides.

Nanomolar concentrations of cathelicidins could induce an oxidative burst that was detected within seconds of exposure, despite the fact that most organisms produce enzymes (catalases, peroxiredoxins, and superoxide dismutases) to counter the destructive effects of harmful oxidants. In Gram-negative bacteria, low micromolar concentrations of B22 or B22a overwhelmed antioxidation defenses and killed the cells. Pretreatment with the iron chelator DP or hydroxyl radial scavenger TU increased cell survival after exposure to B22, and overexpression of the superoxide dismutase SodA completely countered the killing effects of the peptide in V. cholerae. In Gram-positive bacteria, similar observations were reported following treatment of S. aureus with human β-defensin 3 (35). Strikingly, at higher concentrations, cathelicidin treatment caused membrane-bound blebs containing displaced cytoplasmic material to bud from cells and leave behind empty peptidoglycan remnants, neither of which was viable.

**FIG 11:**
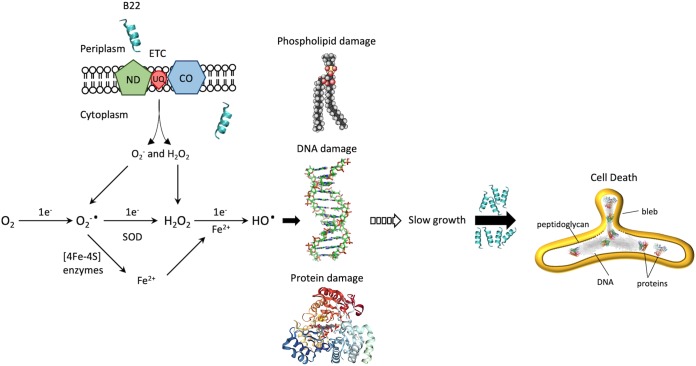
Model for the mechanism of B22-mediated killing. Cathelicidin B22 rapidly binds to cells and permeabilizes the cell membrane, disrupting the membrane-bound aerobic respiratory electron transport chain (ETC) and degrading the proton motive force in such a way that superoxide (O_2_^−^) is released from the complex. The scavenging by O_2_ of an electron from flavin cofactors in the respiratory chain of aerobically respiring bacteria or reduced cytoplasmic flavins provides another source of O_2_^−^ and H_2_O_2_. O_2_^−^ is normally converted to hydrogen peroxide (H_2_O_2_) by superoxide dismutase (SOD). However, Fe^2+^ can be leached by free O_2_^−^ from cytoplasmic enzymes with [4Fe-4S] clusters and can react with H_2_O_2_ to produce ^·^OH that damages cellular components, including DNA, membrane lipids, and proteins. This can slow cell growth at nanomolar concentrations and lead to cell death at micromolar concentrations. ND, NADH dehydrogenase; CO, cytochrome oxidase; UQ, ubiquinone.

Our data suggested that engineered cathelicidins have a common mechanism of action on Gram-negative and Gram-positive cells, despite the fact that higher concentrations of cathelicidins were needed to inhibit Gram-positive bacteria. For Gram-negative bacteria, we propose that the peptides rapidly bind to cells and interact with membrane lipids (Fig. 11). In doing so, they perturb the cell membrane and disrupt the membrane-bound aerobic respiratory electron transport chain, degrading the proton motive force in such a way that O_2_^−^ is released from the complex. The scavenging by O_2_ of an electron from flavin cofactors in the respiratory chain of aerobically respiring bacteria or reduced cytoplasmic flavins provides another source of O_2_^−^ and H_2_O_2_ (31). Fe^2+^ leached by O_2_^−^ from cytoplasmic enzymes with [4Fe–4S] clusters can react with H_2_O_2_ to produce ^·^OH that damages cellular components, including DNA, membrane lipids, and proteins (33, 36). Indeed, some of the most oxidized proteins include those bearing iron or divalent cation-binding sites, such as glutamate synthase, pyruvate kinase, PtsI, peptide chain elongation EF-Tu and EF-G, chaperones GroEL and DnaK, amino acid biosynthesis and nitrogen assimilation (GlnA, GltD), and universal stress protection (Usp) proteins (37). These enzymes participate in the tricarboxylic acid (TCA) cycle, the pentose phosphate pathway, and other key biosynthetic pathways required for growth (38, 39). Functional inhibition can slow cell growth at nanomolar concentrations and lead to cell death at micromolar concentrations.

The ROS that are induced by B22 and other cathelicidins (30) are likely to interfere with a wide range of molecules, such as lipids, membrane and cytoplasmic proteins, and DNA. The general damage resulting from peptide treatment would be difficult for bacteria to overcome by mutation of target molecules, since multiple essential biological pathways are simultaneously affected. Rather, resistance mechanisms that alter membrane permeability or that sequester, degrade, or efflux the peptides (40) might evolve. Examples of these include the LPS glycylation system identified in V. cholerae El-Tor isolates (41), PagP-mediated lipid A acylation (42, 43), and periplasmic peptide-binding proteins (44). However, we note that the truncated cathelicidins described here have significant penetration power. They killed colistin-resistant bacteria with acetylated lipopolysaccharides that render the membrane refractory to polymyxins. They killed clinical isolates of K. pneumoniae that produce an abundance of extracellular polysaccharides. They killed a V. cholerae El-Tor isolate with amino acid modifications of the LPS. They also penetrated the extracellular matrix of bacterial biofilms and the thick cell wall of Gram-positive bacteria.

The majority of studies on the bacteriostatic/cidal effects of AMPs utilize membrane saturating concentrations (micromolar) that are not physiologically relevant. Notably, LL-37 was previously shown to strongly inhibit P. aeruginosa (45) and Staphylococcus epidermidis (46) biofilms at concentrations well below the MIC, and exposure of E. coli to sublethal micromolar concentrations of antimicrobial peptides imposed a division block that induced cell filamentation (47). We have shown that nanomolar concentrations of B22 and B22a slowed growth of Gram-negative and Gram-positive bacteria. We propose that low circulating levels of AMPs may serve to slow bacterial growth and lengthen the window for an immune response. Given the role of cathelicidins in modulating inflammatory responses, it is tempting to speculate that their bacteriostatic activity at lower concentrations coevolved with immune function.

## MATERIALS AND METHODS

### Media and strains.

Bacteria were cultured in cation-adjusted Mueller-Hinton broth (CAMHB) overnight before antimicrobial testing. The majority of the strains were obtained from Karen Bush. Human red blood cells (hRBCs) were purchased from Innovative Research, Inc. (Novi, MI).

### Peptides.

All peptides were custom synthesized by Ontores Biotechnologies (Zhejiang, China) to a purity of >95%. The masses and sequences of the peptides were verified by mass spectrometry. All other antibiotics were purchased from Sigma-Aldrich (St. Louis, MO).

### Antimicrobial activity assays.

MICs were determined by using the broth microdilution method based on the general recommendations of the Clinical and Laboratory Standards Institute (CLSI). Bacteria were grown in CAMHB overnight. They were then diluted into fresh medium the following morning and grown to early log phase. The cultures were then diluted to approximately 5 × 10^5^ CFU ml^−1^, and 100 μl was added to sterile 96-well polypropylene microtiter plates (Corning, NY, USA) preloaded with twofold dilutions of antimicrobial peptides. The plates were incubated for 16 to 18 h, and the lowest peptide concentration required to completely inhibit bacterial growth was recorded. All MIC assays were determined from at least three independent assays.

The numbers of CFU were determined using 5 × 10^5^ CFU/ml of early log phase bacteria that were incubated in CAMHB with 2 μM peptide for 1 h at the optimal growth temperature. Bacteria were then diluted and spread onto agar plates for overnight growth to determine the number of CFU. The effects of peptides on bacterial growth were determined by diluting early-log-phase cultures in CAMHB to an optical density at 600 nm (OD_600_) of approximately 0.05. Then 100 μl of the culture was added to 96-well plates and incubated at the optimal growth temperature with continuous shaking in a Biotek Synergy H1 plate reader (Biotek Inc.). The optical density was monitored every 2 min for 1 h, the peptides were added to the indicated concentration, and bacterial growth was monitored for another 90 to 120 min. All growth rates shown are the average of three independent trials.

### Cell cytotoxicity assays.

The hemolytic activities of peptides were determined using human red blood cells (hRBCs). The hRBCs were washed three times with phosphate-buffered saline (PBS) (pH 7.4) and then resuspended in PBS. The hRBC solution was mixed with serial dilutions of peptides in PBS buffer, and the reaction mixtures were incubated at 37°C for 45 min. After centrifugation at 94 × *g* for 10 min to pellet intact hRBCs, hemoglobin release was monitored by measuring the absorbance of the supernatant at 415 nm. The background level of absorbance was measured for hRBCs incubated with PBS buffer alone. hRBCs incubated with water were used as the reference for 100% hemolysis. The percentage of hemolysis (%Hem) was calculated as follows: %Hem = [(*A*_sample_ − *A*_blank_)/*A*_water_] × 100 where *A*_sample_ is the absorbance of the sample, *A*_blank_ is the absorbance of the blank, and *A*_water_ is the absorbance of water.

### Fluorescence microscopy.

Single-cell static, time-lapse images and movies were captured on an Olympus IX83 inverted microscope using a 100×, 1.3-numerical-aperture phase-contrast objective. Phase-contrast and fluorescence images were obtained with a Hamamatsu ORCA-R2 digital charge-coupled-device camera, and the light source was the Xcite 120 light-emitting diode (Lumen Dynamics, Mississauga, ON, Canada). Emission filters were purchased from Chroma Technology (Bellows Falls, VT). The specific emission filters were DAPI-5060C-OMF (excitation [EX] filter, 377/50 nm; emission [EM] filter, 447/60 nm; dichroic mirror [DM], 409 nm), GFP- 3035D-OMF (EX filter, 473/31 nm; EM filter, 520/35 nm; DM, 495 nm), mCherry-B-OFF (EX filter, 562/40 nm; EM filter, 641/75 nm; DM, 593 nm). Images were processed with the Olympus software package cellSense Dimensions (v 1.14). Data from three biological replicates were analyzed for each strain. Images presented are from a single representative experiment.

### Microfluidic flow cells.

Imaging of individual cells was conducted using the CellAsic microfluidic perfusion system (ONIX) with integrated temperature controller and B04A bacterial microfluidic plates (EMD Millipore, Billerica, MA). The CellAsic ONIX FG software (v 5.0.2.0) was used to control flow rate and deliver fresh medium with or without antimicrobial peptide (AMP). The chambers were loaded by perfusion of 50 μl of a 10^6^ cells/ml bacterial suspension at 2 lb/in^2^ for 15 s to prime the cells followed by 4 lb/in^2^ for 15 s to trap the cells. The chamber was then rinsed at 1 lb/in^2^ for 30 s followed by 5 lb/in^2^ for 5 min. To quickly switch solutions, medium was perfused at 10 lb/in^2^ for 10 s, and then flow was reduced to 2 lb/in^2^ for the remainder of the imaging.

To monitor biofilm formation, polydimethylsiloxane (PDMS)-glass flow cell devices containing eight chambers (40 by 5 by 1 mm) were fabricated as previously described (48). The chambers were sterilized by sequential treatment with 50 ml each of 3% H_2_O_2_, sterile H_2_O, and CAMHB prior to inoculation. Mid-log cultures (OD_600_ of 0.1) were seeded into separate flow cell chambers. Initial attachment (no flow) proceeded for 30 min followed by a flow rate of 0.5 ml min^−1^ for 4 h in medium containing a 20,000-fold dilution of the LIVE/DEAD *Bac*Light Bacterial Viability kit (ThermoFisher). Flow was then switched to the same medium lacking or containing B22 at the indicated concentration. Time-lapse biofilm images and z-stacks (20 1-μm slices) were captured with an Olympus IX83 microscope using a UPLSAPO 40× silicon oil immersion objective (numerical aperture [NA], 1.25; working distance [WD], 0.3 mm). Quantitative analysis to determine biofilm biomass was performed using cellSense (Olympus) and Comstat (49). Data from three biological replicates were analyzed for each strain. Images presented are from a single representative experiment.

### Assay for peptide-induced membrane damage.

SYTOX Green (Invitrogen, Carlsbad, CA, USA) uptake was used to monitor the effect of the AMPs on bacterial membranes. Mid-log cells were resuspended in CAMHB to 1 × 10^6^ CFU/ml and seeded into microfluidic chambers as described above. The cells were then flushed with medium containing 0.1 μM SYTOX Green for 5 min. The same medium was used for control cells, while the medium for treatment samples also included AMP at the indicated concentration. Fluorescence was detected by microscopy as described earlier using the GFP filter set.

### Assay for ROS production.

Early exponential bacterial cells were seeded into microfluidics chambers as described earlier and washed in fresh medium containing 2.5 μM CellROX Green (ThermoFisher) with or without peptide. CellROX fluorescence was monitored by microscopy using the green fluorescent protein (GFP) filter set. To inhibit Fenton chemistry in the cytoplasm, cells were pretreated with 75 μM concentration of the membrane-permeable agent 2,2′-dipyridyl (DP), which efficiently chelates free Fe^2+^, and 150 μM concentration of the scavenger thiourea (TU), which directly quenches Fenton-generated hydroxyl radicals (50). These concentrations of DP and TU were confirmed not to affect bacterial growth.

### Pulse-chase labeling of peptidoglycan with fluorescent d-amino acids.

The fluorescent d-amino acid (FDAA) NADA (4-chloro-7-nitrobenzofurazan 3-amino-d-alanine) or TADA (tetramethylrhodamine 3-amino-d-alanine) (18, 51) was added to early exponential cells (OD_600_ of 0.05) to a final concentration of 100 μM. Cultures were grown to an OD_600_ of 0.20 and placed on ice for 2 min to halt labeling. One milliliter of culture was centrifuged at 14,000 × *g* for 5 min at 4°C. Cell pellets were washed once with cold medium, resuspended, and seeded into microfluidic chambers at 10°C. Flow was then initiated with prewarmed medium without peptide (control) or with the indicated concentration of peptide (treatment). Where indicated, expression of a FliM-tdTomato fusion (10) was induced with 0.1% l-Ara (Sigma-Aldrich), and cells were stained with 4′,6′-diamidino-2-phenylindole (DAPI) (Sigma-Aldrich) for 5 min prior to the addition of 6-carboxyfluorescein (FAM)-labeled B22.
