# Supplementary figures and images for: Cathelicidin Peptides Restrict Bacterial Growth via Membrane Perturbation and Induction of Reactive Oxygen Species
Source: mBio. 2019 Sep 10;10(5):e02021-19. doi: 10.1128/mBio.02021-19 (PMC6737244; doi:10.1128/mBio.02021-19)

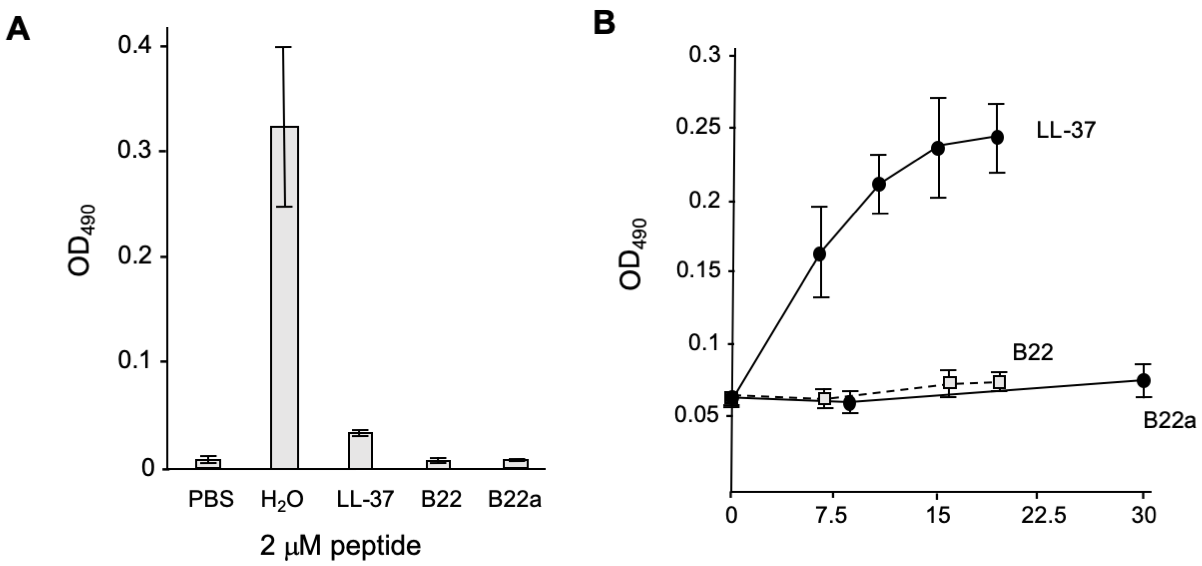

Supplement: FIG S1 [file mBio.02021-19-sf001.tif]

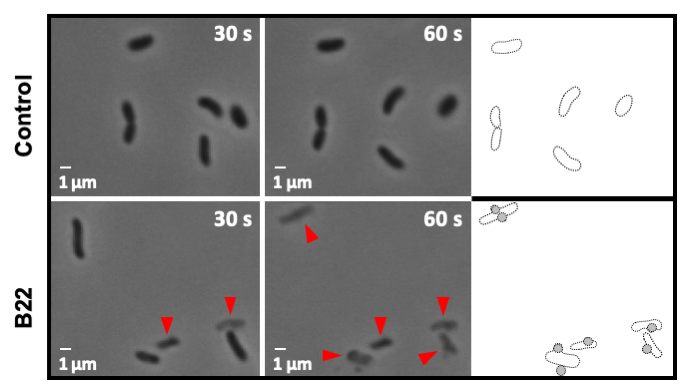

Supplement: FIG S2 [file mBio.02021-19-sf002.tif]

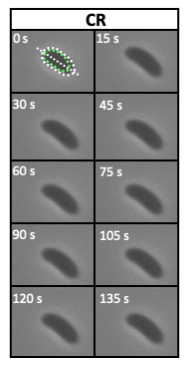

Supplement: FIG S3 [file mBio.02021-19-sf003.tif]

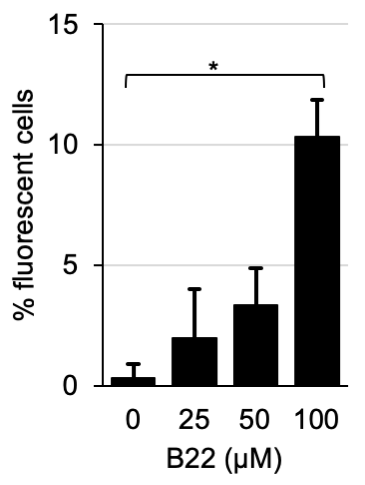

Supplement: FIG S4 [file mBio.02021-19-sf004.tif]

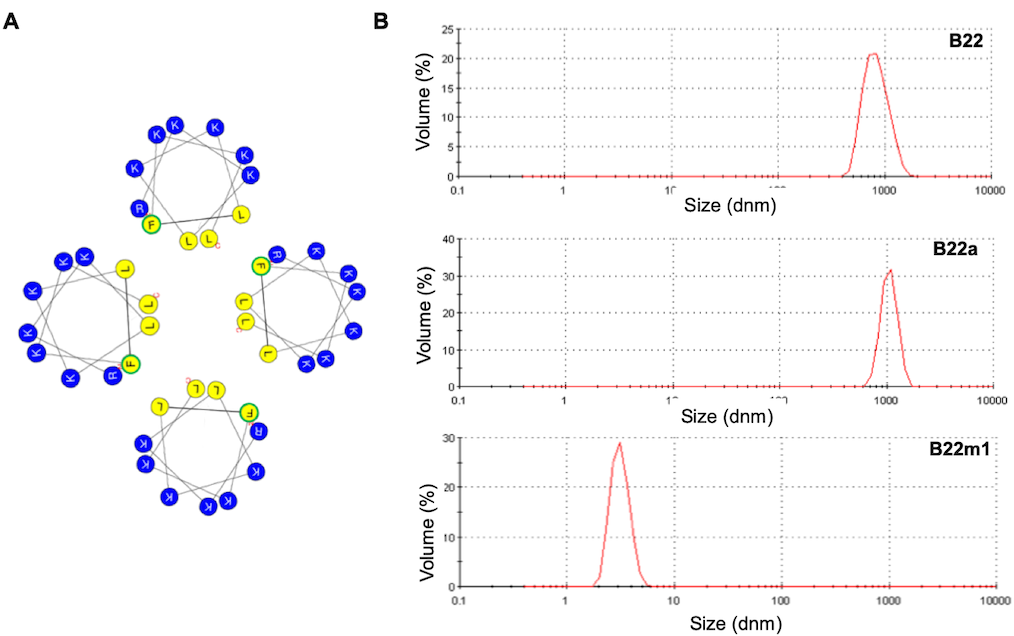

Supplement: FIG S5 [file mBio.02021-19-sf005.tif]
